# Enhancing patient engagement in cancer research: a focus on patient‐centric approaches to scientific discovery

**DOI:** 10.1002/1878-0261.70004

**Published:** 2025-02-21

**Authors:** Estela Cepeda, Marina Reguero, Vanesa Abón, Marta Puyol

**Affiliations:** ^1^ Scientific Foundation of the Spanish Association Against Cancer Madrid Spain

**Keywords:** health equity, patient advocacy, patient barriers, patient‐centered research, population diversity, public engagement

## Abstract

Patient engagement in healthcare and research empowers patients to actively participate in decision‐making, treatment planning, and research design, fostering better treatment outcomes and relevance. However, barriers, such as power imbalances, structural limitations, a lack of awareness, and socioeconomic disparities, often hinder meaningful involvement, particularly for marginalized groups. Overcoming these challenges requires inclusive frameworks and infrastructures to promote collaboration among patients, researchers, and other stakeholders. Initiatives like the Patient Engagement Open Forum or World Cancer Research Day underscore the importance of patient‐centered approaches. By fostering trust, transparency, and shared decision‐making, a more inclusive and impactful healthcare and research ecosystem can be achieved.

## Graphical abstract

Patient engagement involves actively including patients in healthcare decisions and research to ensure care and studies align with their needs. This approach improves outcomes, trust, and communication while fostering collaboration between patients and professionals. Despite progress, barriers like unequal access and lack of awareness remain. Overcoming these challenges requires inclusive partnerships, diverse representation, and a shared commitment to patient‐centered care and research.

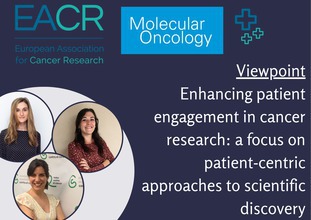

**Abbreviations**
PE: patient engagement
UICC: Union for International Cancer Control

Patient engagement (PE) in health care refers to strategies that actively involve patients in their care, emphasizing shared decision‐making between patients and healthcare providers to make informed choices about their overall health management. Beyond direct care, PE extends to research, where patients contribute their perspectives to shape more relevant and impactful studies. Therefore, there is a growing demand for meaningful patient involvement in research, with increasing efforts from various stakeholders to engage patients in these initiatives.

Patient engagement in decision‐making carries many benefits as it encourages patients to take a proactive role in monitoring their health, understanding treatment options, and adhering to care plans. Moreover, this approach leads to improved personal outcomes by fostering a feeling of belonging in patients and benefits the healthcare system by enhancing patient–scientist/clinician communication, building trust and optimizing resource allocation. By empowering patients and valuing their experiences, PE becomes a cornerstone of a more inclusive, effective, and patient‐centered healthcare system [1].

Patient engagement in biomedical research has been steadily increasing over the past few decades, with a consistent rise in scientific publications in this topic since the 1980s, as shown by an analysis of the PubMed database. Initially, PE initiatives in clinical research were developed mostly to inform and consult patients about research aspects and have progressively advanced to integrate the patient voice in research. These have been particularly significant in the context of chronic conditions like diabetes or neurodegenerative diseases to improve treatment adherence. Some recent examples in diabetes are the T1D Exchange initiative in the United States, that connects researchers and individuals with type 1 diabetes to co‐develop studies and share real‐world data; the DIRECT in Europe (Diabetes Research on Patient‐Centered Strategies, Europe), which is a European consortium that involves patients in the development of precision medicine approaches for diabetes, ensuring treatments are tailored to individual needs; or other global patient advocacy initiatives like the Diabetes Patient Advocacy Coalition. Moreover, in the field of neurodegenerative diseases there are some recent examples, such as the Parkinson's Foundation Research Advocates Program in the United States, which trains patients to become active partners in Parkinson's disease research, ensuring studies reflect patient priorities and real‐world concerns; and the IMI‐PainCare in Europe, a large research initiative involving chronic pain patients, including those with neurodegenerative conditions, to co‐develop treatment strategies and improve clinical trials. Nevertheless, until now similar efforts like those initiatives have remained comparatively scarce in the field of cancer research, although, some activities are expanding in western countries, mostly in clinical research.

Despite its advantages, so far, a patient's voice has been hardly heard during the development of healthcare policies and research strategies due to several reasons:

1. **Power dynamics and cultural resistance**: Hierarchical systems in research and policymaking often prioritize expert knowledge over lived experiences, and institutions tend to undervalue patients as equal collaborators, limiting their meaningful contributions, due to fear of disrupting power dynamics [2].
2. **Structural and communication barriers:** The lack of access to decision‐making platforms, the technical and complex nature of research and policymaking as well as language differences, can discourage patient participation.
3. **Lack of awareness:** Many patients remain unaware of the existence of PE initiatives, which can foster distrust toward scientific institutions.
4. **Tokenism**: When patients are included, their involvement can sometimes be symbolic rather than impactful on decision‐making, many times due to inadequate training of researchers in participatory approaches. Meaning that to enhance patient engagement in research, science should be made more accessible to them. To achieve this, scientists could receive training to communicate research more effectively to a nonscientific public.
5. **Lack of infrastructure and limited resources**: Engaging patients meaningfully requires time, funding, and logistical support that may not be prioritized.
6. **Socioeconomic and demographic barriers**: Marginalized groups often face systemic barriers to engage in research and decision‐making, such as financial constraints, difficulty in accessing patient advocate groups, unequal access to resources and historical discrimination. This situation is particularly critical in low‐ and middle‐income countries.
7. **Perception of patients' role**: Patients are often seen as passive recipients of care rather than active contributors to the system.

Addressing these issues requires intentional efforts from all involved stakeholders to build inclusive frameworks, create equal partnerships, and acknowledge the unique insights that patients bring to the table [3].

Basic and translational researchers often have limited direct interaction with patients compared to clinical researchers. However, even within clinical settings, effectively integrating patients into the research process is challenging. Cancer associations can help by acting as hubs to foster collaboration and meaningful dialog, allowing researchers and patients to co‐design studies that better align with patients' needs and priorities.

To actively collaborate with patients, researchers and healthcare providers must challenge their personal and professional assumptions, be encouraged to rethink representation, and adopt innovative approaches to engaging diverse groups. Moreover, the involved professionals should evaluate the inclusiveness of their work and be open to self‐reflection, integrating these insights into the study's design, execution, and scope.

Thus, a willingness to disassemble the self and deconstruct one's assumptions is essential in an efficient PE in research, ensuring that power is not imposed over individuals or groups, and instead focusing on fostering equitable collaboration between partners.

On this point, to promote significant PE in research, systemic changes are needed, including educating researchers in collaborative methodologies, ensuring diverse patient representation, and building trust between patients and research institutions. In addition to researchers and patients, other stakeholders—healthcare providers, patient advocacy groups, ethics committees and institutional review boards, policymakers and funders, and communities and but also society in general—must be involved [4]. Hence, efforts to promote PE in research should aim to establish a meaningful collaboration between those parties, especially between patients and researchers, where five key principles must be considered (Fig. 1):

- The patient–scientist relationship must be reciprocal, emphasizing mutual respect, shared decision‐making, and collaborative learning where both parties contribute their unique perspectives and expertise to the research process for mutual benefit.
- The research community and the funding institutions must keep patients informed throughout the research process to build trust, ensure transparency, and foster a sense of shared belonging. Keeping patients informed requires intentional efforts from different stakeholders, to communicate clearly and inclusively at every stage of the study.
- Researchers should provide the public and patients with a clear and informative framework to guide their participation in the research process. This helps establish expectations, clarifies roles, and empowers patients to contribute effectively and meaningfully ensuring that their involvement is both impactful and aligned with the study's objectives.
- Communities are diverse and composed of unique subgroups, requiring researchers and funders to embrace this complexity for inclusive and impactful research that reflects true population diversity.
- Patient involvement in research requires more time and empathy because it shifts the traditional dynamic of research from being solely expert driven to a collaborative process. This approach prioritizes understanding patients' lived experiences and needs, building trust, and fostering meaningful participation.

**Fig. 1:**
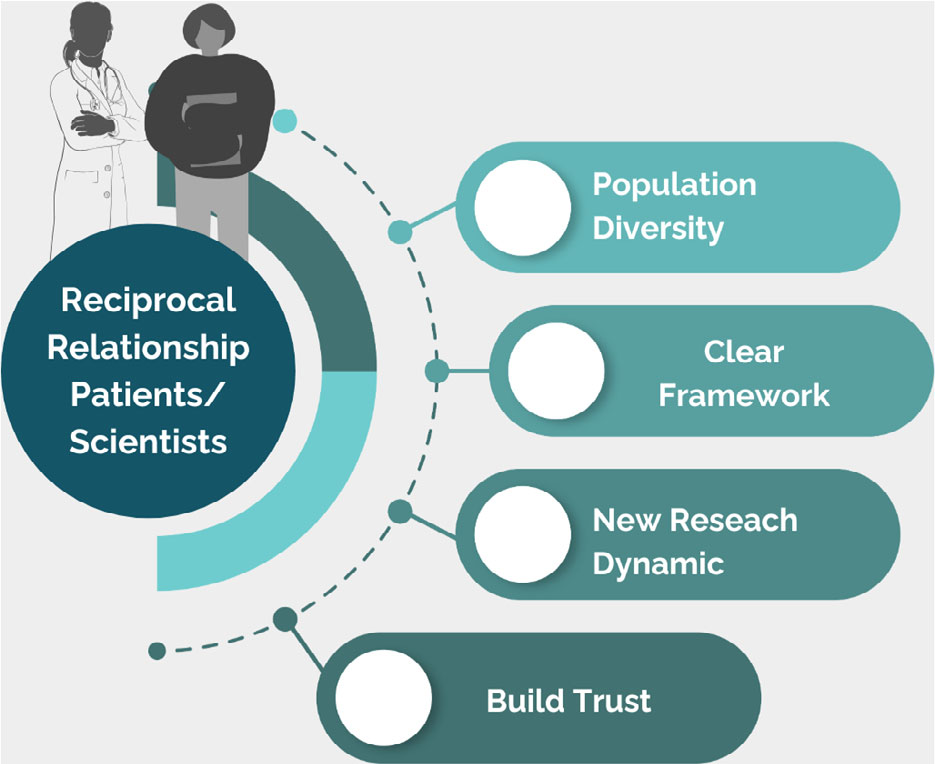
Five key elements to consider when including patient perspective in research. It is essential to foster a collaborative and mutually beneficial partnership between scientists and patients by taking into account patient diversity, establishing a clear framework of action and a new research dynamic, all aim to build trust within the community.

The future of PE in research is shifting toward a more inclusive, collaborative, and patient‐focused model. A growing number of collaborative actions and initiatives have been established to foster PE in health care and research, professionalizing the field such as

- the Patient Engagement Open Forum (https://patientengagement.synapseconnect.org/),
- Patient Focused Medicines Development (https://patientfocusedmedicine.org/),
- the European Patients' Academy of Therapeutic Innovation (https://eupati.eu/about‐us/),
- FDA (Food and Drug Administration) (https://www.fda.gov/patients/learn‐about‐fda‐patient‐engagement).
- Some organizations that promote patient engagement in cancer research are:
  ○ Patdvocates (https://www.patvocates.net/),
  ○ Cancer Patients Europe (https://cancerpatientseurope.org/),
  ○ European society Medical Oncology (ESMO) (https://www.esmo.org/policy/patient‐advocates‐working‐group),
  ○ European organization for research and treatment of cancer (EORTC) (https://www.eortc.org/patient‐involvement/patient‐in‐research/).

Moreover, the World Cancer Day campaign, spearheaded by the Union for International Cancer Control (UICC), underscores the significance of patient‐centered health care through its 2025–2027 theme, ‘United by Unique’. This initiative emphasizes placing people at the core of care while exploring innovative strategies to create meaningful impact. Similarly, the new strategic plan for World Cancer Research Day highlights patient‐centered research as a cornerstone of its mission, aiming to raise awareness of the pivotal role patients play in cancer research and health care.

This evolving landscape demands a paradigm shift in how researchers view the value of integrating patients' perspectives into scientific endeavors, recognizing their potential to enhance both outcomes and overall impact.

Thus, integrated and inclusive models of engagement are needed to support further transformative efforts. On this point, virtual platforms and meetings that bring researchers and patients together offer innovative opportunities to enhance interaction and strengthen the role of PE in research.

Building a truly patient‐centered research ecosystem requires not only inviting diverse voices to the table but also rethinking the current research structures that leave out the patient's voice and the will to overcome the barriers found. Only by working together, through collaboration, trust, and shared goals can we achieve a future where science serves everyone, equitably and meaningfully.

## Conflict of interest

The authors declare no conflict of interest.

## Author contributions

MP conceived the manuscript. MP, EC, and MR wrote the manuscript. VA, EC, MR, and MP revised the manuscript. All the authors read and approved the final manuscript.
